# Intrafamilial Transmission of HTLV-1 and HTLV-2 in Indigenous Peoples of the Brazilian Amazon: Molecular Characterization and Phylogenetic Analysis

**DOI:** 10.3390/v16101525

**Published:** 2024-09-26

**Authors:** Isabella Nogueira Abreu, Felipe Bonfim Freitas, Eliene Rodrigues Putira Sacuena, Gabriel dos Santos Pereira Neto, Bruno José Sarmento Botelho, Carlos Neandro Cordeiro Lima, Vanessa de Oliveira Freitas, Mike Barbosa dos Santos, Sandra Souza Lima, Ricardo Ishak, João Farias Guerreiro, Antonio Carlos Rosário Vallinoto, Izaura Maria Cayres Vallinoto

**Affiliations:** 1 Laboratório de Virologia, Universidade Federal do Pará, Belém 66075-110, Brazil; isabella.nogueira@oulook.com.br (I.N.A.); gabrielnetoenf@gmail.com (G.d.S.P.N.); bruno.botelho@ics.ufpa.br (B.J.S.B.); neandro_lima@live.com (C.N.C.L.); freitas3@outlook.com (V.d.O.F.); sandra.souza.lima@gmail.com (S.S.L.); rishak@ufpa.br (R.I.); ivallinoto@ufpa.br (I.M.C.V.); 2 Seção de Virologia, Instituto Evandro Chagas, Ananindeua 67030-000, Brazil; felipebonfim@iec.gov.br; 3 Laboratório de Genética Humana e Médica, Universidade Federal do Pará, Belém 66075-110, Brazil; putirasacuena@gmail.com (E.R.P.S.); joaofg@ufpa.br (J.F.G.); 4 Seção de Arbovirologia e Febres Hemorrágicas, Instituto Evandro Chagas, Ananindeua 67030-000, Brazil; psykenett@hotmail.com

**Keywords:** HTLV, indigenous people, Brazilian Amazon, intrafamily transmission, phylogeny

## Abstract

Human T-limphotropic virus 1 infection has a global distribution, with a high prevalence in some regions of Brazil and the world, while HTLV-2 infection is endemic mainly among indigenous people and drug users. To analyze intrafamilial transmission of HTLV-1/2 in five Kayapó indigenous peoples (Gorotire, Kararaô, Kokraimoro, Kubenkokre, and Xikrin do Bacajá), we investigated 1452 individuals who underwent serological and molecular tests. Among the 276 indigenous people with positive results, we identified intrafamily transmission in 42.7% of cases, representing 38 families. It was possible to suggest horizontal and vertical transmissions in 15.8% (6/38) and 47.4% (18/38) of the family groups, respectively. In 15.8%, it was not possible to suggest the route, which indicated that the transmission may have occurred through both vertical and horizontal routes. Through phylogenetic analyses, 35 samples positive for HTLV-2 were sequenced and classified as subtype 2c, and the two samples that tested positive for HTLV-1 were shown to belong to the cosmopolitan subtype, transcontinental subgroup (HTLV-1aA). This study confirms the intrafamilial transmission of HTLV-1/2 infection in indigenous people of the Brazilian Amazon, highlighting the importance of the sexual and mother-to-child transmission routes in maintaining the virus in these people.

## 1. Introduction

Human T-lymphotropic viruses 1 and 2 (HTLV-1 and HTLV-2) belong to the *Retroviridae* family and the *Deltaretrovirus* genus [1]. This family is associated with the development of lymphomas, sarcomas, leukemias, and hematological, inflammatory, and neurological diseases [2,3].

HTLV-1/2 infection is still considered neglected today, mainly because, for a long time, it was assumed to have a low impact on public health, given that the majority of infected individuals do not present symptoms of diseases associated with the virus, and due to its greater frequency of occurrence in countries and regions with low per capita income [4].

It is estimated that only 1 to 5% of people living with HTLV (PLWH), especially those infected with HTLV-1, may develop diseases such as HTLV-1-associated myelopathy (HAM) [5,6] and adult T-cell leukemia/lymphoma (ATLL) [7,8] and other inflammatory clinical manifestations, such as bronchiolitis, bronchitis, bronchiectasis [9], uveitis [10]. Other diseases, such as arthritis [11], infectious dermatitis [12,13], Sjogren’s syndrome [14], neurogenic bladder [15], and erectile dysfunction [16], have also been associated with HTLV-1 infection. Furthermore, some opportunistic infections, such as disseminated *Strongyloides stercoralis* [17] and *Mycobacterium tuberculosis* [18] infections, are also frequently observed to be associated with HTLV-1 infection. On the other hand, unlike HTLV-1 infection, HTLV-2 infection is considered predominantly asymptomatic, with rare cases associated with neurological disease [19,20,21,22,23,24] and, more recently, with fibromyalgia [25].

These viruses can be transmitted sexually, with the most effective transmission occurring from men to women [26,27,28], or they can spread via vertical transmission from mother to child during pregnancy, through the transplacental route, vaginal birth, or breastfeeding [29,30,31]. Among these forms, breastfeeding is the main form of transmission, especially over prolonged periods of more than six months, as the lymphocytes present in breast milk are transferred to the baby during breastfeeding [32,33,34,35]. (3) The viruses can be transmitted through the use of contaminated sharps, such as syringes and needles, by intravenous drug users [36,37] or through the transfusion of blood products [38,39,40]. However, countries such as Japan, Saint Lucia, Chile, the USA, Canada, Brazil, and many European countries have already implemented screening measures in blood banks, making this form of transmission less common in these locations [41,42,43,44].

It is estimated that 10 to 20 million people are living with HTLV-1 worldwide. However, the virus is considered endemic in certain regions, such as Japan, Africa, the Caribbean islands, Central and South America, and Central Australia [45,46]. In South America, Brazil stands out as the country with the greatest absolute number of people living with HTLV, with north and northeast regions having the highest rates [47]. In particular, Bahia is the state with the highest prevalence of HTLV-1 infection, and Pará is the state with its significant occurrence among indigenous populations [48,49,50].

Considering the silent and neglected nature of the infection, as well as the high number of cases, especially among Indigenous peoples of the Brazilian Amazon, the present study aimed to investigate the intrafamilial transmission routes that contribute to the spread and maintenance of the virus in populations of Indigenous people in which the virus is hyperendemic.

## 2. Materials and Methods

### 2.1. Study Population

The study population included men and women ranging from 2 months to 94 years old, totaling 1452 individuals belonging to five indigenous peoples (Gorotire, Kararaô, Kokraimoro, Kubenkokre, and Xikrin do Bacajá) of the Kayapó ethnic group, which is located in the Brazilian Amazon region, as described by Abreu et al. [50].

### 2.2. Serological and Molecular Analysis

All samples were analyzed via a serological ELISA (MUREX HTLV-I + II, DiaSorin, Dartford, UK), and subsequently, the reagent samples were subjected to Western blot analysis (HTLV Blot 2.4 kit—MP Diagnostics, Singapore), INNO-LIA HTLV-I/II (Fujirebio, Tokyo, Japan) and real-time PCR (qPCR) [51] for confirmation and differentiation of the viral type [50].

### 2.3. Investigation of Intrafamily Transmission

The search for intrafamilial transmission (horizontal and vertical) of HTLV-1/2 infection was carried out by analyzing family groups of individuals who were confirmed to be positive for HTLV-1/2 infection. Subsequently, using the demographic censuses of each Indigenous community provided by the Special Indigenous Health District (DSEI), we identified the degree of kinship of the individuals (mother, father, spouses, and children), thus allowing us to suggest possible transmission routes within the same family. In total, 302 people belonging to 38 families were investigated.

### 2.4. Amplification of the 5′LTR Region and Sequencing 

Samples that tested positive for HTLV-1/2 were subjected to amplification of the 5′LTR region to identify the molecular subtype. The reaction mixture contained 15.95 µL of ultrapure water, 1.25 µL of buffer (10×), 1.5 µL of MgCl_2_ (50 mM), 3.0 µL of dNTPs (10 mM), 0.5 µL of each primer (20 pmol), 0.3 µL of Taq (1 U/µL) and 2.0 µL of DNA. For the first round of amplification for HTLV-1 (844 bp), the primers used were P3LTR (5′-TGACAATGACCATGAGCCCCA-3′) and LTR6 (5′-ATGCCGCCTGGAGGAAGTTAAGC-3′), with the following thermocycling conditions: 94 °C for 5 min, followed by 35 cycles of 94 °C for 30 s, 61 °C for 30 s, and 72 °C for 40 s and 72 °C for 10 min. For the second round, the primers LTR1 (5′-ACCATGAGCCCCAAATATCCCCC-3′) and RU5L (5′-CGGGAAAAGATTTGGCCCATTG-3′) were used, following the same thermocycling conditions but with a primer hybridization step, which was carried out at 60 °C.

For the first round of amplification for HTLV-2 (788 bp), the primers FIILTR (5′-TCGCGATGACAATGGCGACTAGCCTC-3′) and Long Gag (5′-GGGGGCTTTGGGTATTGGAGTTGG-3′) were used; the primers Mo16 (5′-GCCTCCCAAGCCAGCCAC-3′) and MSW gag (5′-GGGAAAGCCCGTGGATTTGCCCCAT-3′) were used for the second reaction. The thermocycling conditions used were as follows: 95 °C for 5 min, followed by 35 cycles of 95 °C for 30 s, 63 °C for 30 s, and 72 °C for 40 s and 72 °C for 10 min.

After amplification of the 5′LTR region, the PCR product (630 bp) was purified and sequenced by the Sanger method using the BigDye Terminator v3.1 sequencing kit (Thermo Fisher, Waltham, MA, USA) and the Genetic Analyzer 3130xl (Applied Biosystems, Westminster, CO, USA) [52].

### 2.5. Nucleotide Sequence Analysis

The nucleotide sequences were analyzed using the Chromas program 2.6.6v (Technelysium—DNA Sequencing Software), in which each sequence underwent a quality analysis using the Phred algorithm, with a minimum value of 30 to ensure 99.9% sequencing accuracy.

The assembly of contigs (a combination of sense and antisense sequences) was performed using BioEdit software 7.2.5v (Biological Sequence Alignment Editor) [53]. The alignment of the sequences of the 5′LTR region was carried out using Geneious Prime software (www.genious.com, Biomatters, Auckland, New Zealand) [54].

For phylogenetic analysis, reference strains available in GenBank were used, representing the HTLV-1 and HTLV-2 subtypes, in addition to global sequences, including strains from different regions of Brazil.

To construct the phylogenetic trees, we used the maximum likelihood (ML) method through MEGA11 software [55] from initial neighbor-joining (NJ) trees using the Tamura-Nei (TN) model. The model included nucleotide substitutions (transitions and transversions), as well as gamma distribution rates between sites with a gamma parameter, a homogeneous pattern between lineages, and partial deletion with a 95% cutoff as initial trees. ML trees were generated using TN G+I with five discrete gamma categories, and gaps were treated with partial deletion at a 95% cutoff. The heuristic method consisted of extensive pruning of subtrees and moderate branch grafting. To infer the bootstrap values, 1000 bootstrap-to-tree interactions were performed. Layout changes were made using FigTree 1.4.4 software (http://tree.bio.ed.ac.uk/software/figtree/; accessed on 30 August 2024).

In addition to phylogenetic analysis, the nucleotide sequences were subjected to similarity analysis. At this stage, similarities were calculated based on the genetic distance between populations from different peoples and specific families. Furthermore, the sequences were paired to investigate the epidemiological characteristics of the distribution of the viral infection using MEGA 11 software. The strains used in the phylogeny and their access codes in GenBank were as follows: BRPA_9_Kubenkokre (PQ339013); BRPA_57_2020_Xic_Bac (PQ339014); BRPA_58_2020_Xik_Bac (PQ339015); BRPA_59_Kubenkokre (PQ339016); BRPA_60_Kubenkokre (PQ339017); BRPA_61_Kubenkokre (PQ339018); BRPA_62_Kubenkokre (PQ339019); BRPA_62_2020_Xik_Bac (PQ339020); BRPA_64_2020_Xik_Bac (PQ339021); BRPA_64_2019_Xik_Bac (PQ339022); BRPA_77_Kubenkokre (PQ339023); BRPA_81_Gorotire_R (PQ339024); BRPA_83_Gorotire_R (PQ339025); BRPA_85_Gorotire_R (PQ339026); BRPA_90_Gorotire_R (PQ339027); BRPA_92_Gorotire_R (PQ339028); BRPA_93_Kokraimoro (PQ339029); BRPA_97_Kokraimoro (PQ339030); BRPA_99_2020_Xik_Bac (PQ339031); BRPA_100_Gorotire_T (PQ339032); BRPA_100_Kokraimoro (PQ339033); BRPA_107_Kokraimoro (PQ339034); BRPA_108_Gorotire_T (PQ339035); BRPA_110_2021_Xik_Bac (PQ339036); BRPA_113_2021_Xik_Bac (PQ339037); BRPA_153_2021_Xik_Bac (PQ339038); BRPA_154_2021_Xik_Bac (PQ339039); BRPA_164_Kubenkokre (PQ339040); BRPA_192_2021_Xik_Bac (PQ339041); BRPA_193_2021_Xik_Bac (PQ339042); BRPA_194_2021_Xik_Bac (PQ339043); BRPA_194_Kubenkokre (PQ339044); BRPA_198_2021_Xik_Bac (PQ339045); BRPA_224_2021_Xik_Bac (PQ339046); BRPA_225_2021_Xik_Bac (PQ339047); BRPA_69_Gorotire_R (PQ339048); BRPA_78_Gorotire_R (PQ339049).

### 2.6. Statistical Analysis

The characteristics of the population were described through descriptive analyses. To identify an association between epidemiological characteristics and HTLV-1/2 infection, Pearson’s chi-square test, G test, and binomial test were applied, considering a significance level of 5% (*p*-value < 0.05). All analyses were performed in BioEstat version 5.3.

## 3. Results

### 3.1. Serological and Molecular Analyses

Among the 1452 individuals tested, 276 (19.0%) were confirmed to have HTLV-1 or HTLV-2 infection. Among the confirmed cases, 0.7% (2/276) of individuals were identified to have HTLV-1 infection, while 97.5% (269/276) were confirmed to have HTLV-2 infection. In 1.8% (5/276) of the samples, it was not possible to confirm the viral type, as the virus was classified only as HTLV (Table 1).

### 3.2. Population Characteristics and Intrafamilial Transmission

In total, 845 women and 607 men were included in the study, with a mean age of 26.9 years (SD ± 20.1) and an age range of 0 to 94 years, from five indigenous peoples of the Kayapó ethnic group (Gorotire, Kararaô, Kokraimoro, Kubenkokre and Xikrin of Bacajá).

Among the positive individuals, the ages ranged from 2 to 94 years (mean = 41.7 years) (SD ± 22.4) for the 180 women and from 2 to 86 years (mean = 42.8 years) (SD ± 24.7) among the 96 male individuals (*p*= 0.0105), with the age group over 61 years being the most affected in both sexes (61/104). Table 2 shows the prevalence of infection according to age group and sex, demonstrating a gradual increase in infection as age advanced.

**Figure 1:**
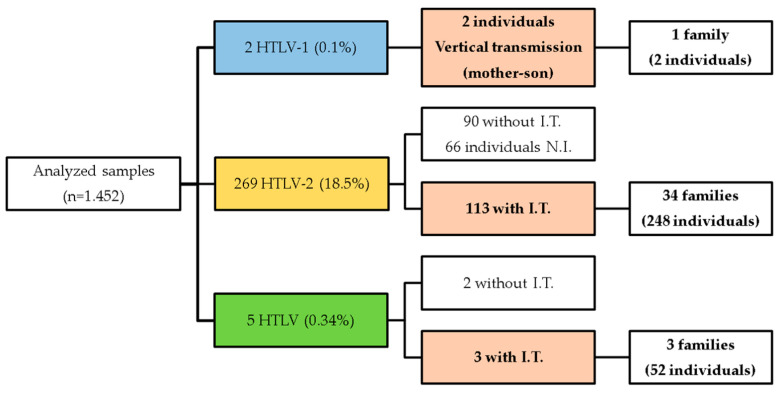
A flowchart of the analysis of the individuals investigated highlights the viral type and the occurrence of intrafamily transmission. I.T.—Intrafamily transmission; N.I.—No information.

According to the investigation of intrafamilial transmission, the two individuals infected with HTLV-1 belonged to the same family (mother and child). Among the 0.3% (5/276) of individuals with a positive result for the HTLV pattern, two did not present intrafamilial transmission. For HTLV-2, out of the total of 18.5% (269/1.452) of positive cases, intrafamilial transmission was observed in 42.0% (113/269), 33.5% (90/269) did not show transmission to other family members, and in 24.5% (66/269), it was not possible to obtain information about family members, making it impossible to assess the likely routes of transmission (Figure 1).

However, intrafamily transmission could be suggested to occur in 42.7% of cases (118 individuals). From these 118 individuals, 184 family members were identified, including parents, children, and spouses, totaling 38 families with 302 indigenous people, including 158 women and 144 men. Of these, 30.1% (91/302) tested negative for HTLV, 39.1% (118/302) tested positive for HTLV, and 30.8% (93/302) were not tested. Of the 118 individuals with HTLV, 62.7% (74/118) were female, with ages ranging from two to 81 years, and 37.3% (44/118) were male, with ages ranging from two to 84 years.

**Figure 2:**
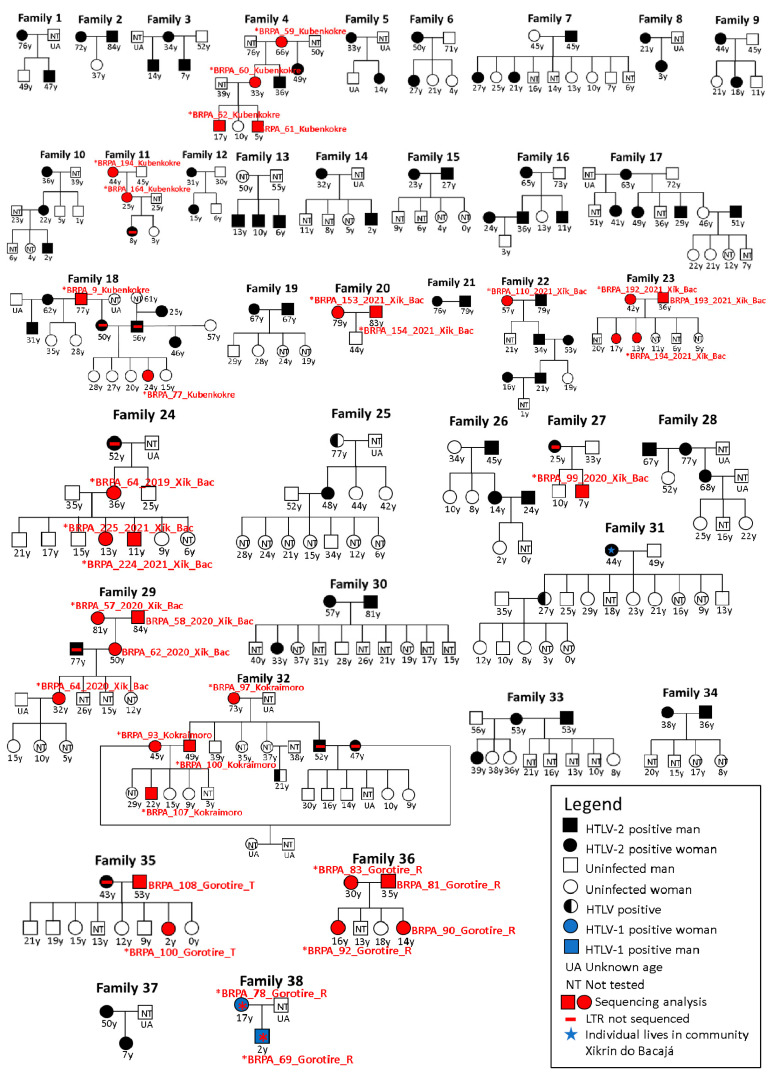
Pedigree showing the possible routes of horizontal transmission (Families 2, 15, 19, 20, 21, and 34), vertical transmission (Families 1, 3–6, 8–12, 14, 17, 24, 25, 27, 31, 37 and 38), vertical and horizontal transmission (Families 23, 28, 30, 33, 35 and 36), vertical or horizontal transmission (Families 16, 18, 22, 26, 29 and 32) and uncommon cases (Families 7 and 13) of HTLV-1 and HTLV-2 among the indigenous peoples Kubenkokre (Families 1 to 18), Xikrin do Bacajá (Families 19 to 30), Kararaô (Family 31), Kokraimoro (Family 32) and Gorotire (Families 33 to 38) of the Kayapó ethnic group located in the Brazilian Amazon.

Figure 2 shows the presence of the virus in the same family group, with the occurrence of infection varying from two to eight people. The infection rates among family members ranged from 11.8% (in two of the 17 members of family 31) to 100% (in family 21, a single family unit with two members). The intrahousehold transmission was observed in the five peoples analyzed, with the highest transmission rate recorded in the Kubenkokre community, in which 55 individuals tested positive across 18 family groups. This was followed by the people of Xikrin do Bacajá (38 infected from 12 families), Gorotire (16 infected from six families), Kokraimoro (seven infected from one family), and Kararaô (two infected from one family).

Among a total of 38 families, isolated cases of horizontal transmission were suggested in 15.8% (6/38) of couples, wherein husbands and wives had the virus, while their children were not infected or were not tested for the virus, as suggested in families 2, 15, 19, 20, 21 and 34. In another 15.8% (6/38) of the families (families 16, 18, 22, 26, 29, and 32), both vertical and horizontal transmission routes were suggested. Figure 2 shows that in four of these families (families 23, 30, 35, and 36), both parents and sons were positive, resulting in transmission to a total of six daughters. In two other families (families 28 and 33), the positive mothers had two marriages. In the first marriage, in which the husband was also positive, there was no vertical transmission. In addition, in the second marriage, where the husbands were negative or not tested, transmission occurred to a daughter in both families. In 47.4% (18/38) of the patients, only vertical transmission was observed, that is, with positive mothers and negative fathers or fathers who were not tested. These families included 24 mothers and 77 children. Among these children, transmission occurred in 37.7% (29/77), including 12 males and 17 females, while 37.7% (29/77) were negative and 24.6% (19/77) were not tested (families 1, 3–6, 8–12, 14, 17, 24, 25, 27, 31, 37 and 38).

In 15.8% of the families (6/38), it was not possible to suggest the type of transmission, i.e., horizontal or vertical, when positive spouses were observed, one of whom was the child of a positive mother. In eight cases, individuals with at least one positive genitor were married to positive partners (families 16, 18, 22, 26, 29, and 32). Therefore, it was not possible to suggest how the transmission occurred. Two unusual cases (5.2%) were observed in two families. In family seven, two daughters, whose father was positive but whose mother was negative, tested positive for the infection. In family 13, three brothers tested positive, but both the mother and father were not tested for HTLV.

### 3.3. Risk Factors Associated with Infection

Table 3 presents the risk factors associated with HTLV-1/2 infection. Of the 1452 individuals investigated, only 761 responded to the epidemiological questionnaire. Notably, children under 10 years of age and individuals from the Kararaô community did not complete the questionnaire.

Among the risk factors presented, it is notable that most individuals with the infection stated that they did not have piercings and did not receive a blood transfusion. When asked about having been breastfed in childhood, 92.2% said yes. Regarding active sexual activity, 25% of individuals responded that they started before the age of 17. Furthermore, 79.7% had an active sex life, with 91.5% having only one sexual partner and only 1.9% using condoms. Table 4 highlights the epidemiological characteristics of women only, including gestational rate and breastfeeding time. The majority (85.9%) of women had children, with 96.2% reporting having breastfed their children for more than six months and 11.0% reporting having breastfed other children in the village. Most women also reported having one to three children (51.0%); however, 17.7% had more than seven children.

In Figure 2, among the families investigated, family groups 1 to 18 correspond to those belonging to the Kubenkokre community, totaling 124 individuals (60 men and 64 women), with vertical transmission being more frequent in 12 of these groups. Nineteen women stated that they had between 1 and 12 children, all of whom were breastfed for more than six months, except for one woman in family 9 whose daughter also carried HTLV-2, even without having been breastfed. Furthermore, six women claimed to have breastfed other children in the village. Regarding sexual transmission, five couples (families 2, 15, 16, and 18) were positive for HTLV-2; all of them had been breastfed in childhood, had only one sexual partner, did not use condoms, had no piercings and had no history of blood transfusions. In family seven, the members who tested positive denied having piercings and having received transfusions. All of them were sexually active.

Families 19 to 30, corresponding to the Xikrin do Bacajá people, had a total of 98 members (46 men and 52 women). Nine of these families (19 to 23, 26, 28 to 30) presented 12 positive couples with similar characteristics with regard to piercings, breastfeeding in childhood, and having a sexual partner (except for one person who chose not to answer this question); these couples did not use condoms, and three of them reported not receiving a transfusion, while six did not respond to this question. The four women who reported having children all breastfed for more than six months and had between one and eight children.

Family 31 corresponds to the Kararaô group, with 17 members (6 men and 11 women). A 44-year-old woman reported not wearing piercings, not having received a blood transfusion, having a sexual partner who did not use a condom, having been breastfed as a child, and having nine children (6 women and 3 men) who were breastfed for more than six months. Six children were tested for HTLV (4 women and 2 men), with one daughter testing positive and the others testing negative. Notably, a 27-year-old positive woman had a 35-year-old husband with an indeterminate result.

The Kokraimoro group, represented by family 32, had 24 members, including 13 men and 11 women. A 73-year-old woman reported not having piercings and not having received a blood transfusion, in addition to stating that she was breastfed as a child and did not maintain an active sexual life. She had 10 children, all of whom were breastfed for more than six months as infants. Two sons and their wives tested positive. The couple denied using piercings and having received transfusions. Both had a monogamous relationship and did not use condoms. Both wives claimed to have breastfed their children for more than six months, in addition to having also breastfed other children in the village. In one of the families, a 22-year-old boy tested positive. When asked about the risk factors related to the infection, he mentioned being sexually active since the age of 15, having three or more partners, and not using condoms.

Families 33 to 38 correspond to the Gorotire people, with 39 individuals (19 men and 20 women). All positive individuals over 16 years of age confirmed that they did not wear piercings, had not received a blood transfusion, and had been breastfed in childhood. The majority mentioned having only 1 sexual partner, except for the 53-year-old man from family 35, who reported having two partners. No participant reported using condoms. All of the positive women older than 16 years old had children and breastfed for more than six months, except for the 17-year-old woman from family 38, who breastfed for less than six months.

Among the positive individuals without intrafamilial transmission, 6.9% reported having piercings, 4.2% received a blood transfusion, 88.9% were breastfed as children, and the majority were sexually active (75.0%), but only 2.8% used condoms.

### 3.4. Phylogenetic Analyses

**Figure 3:**
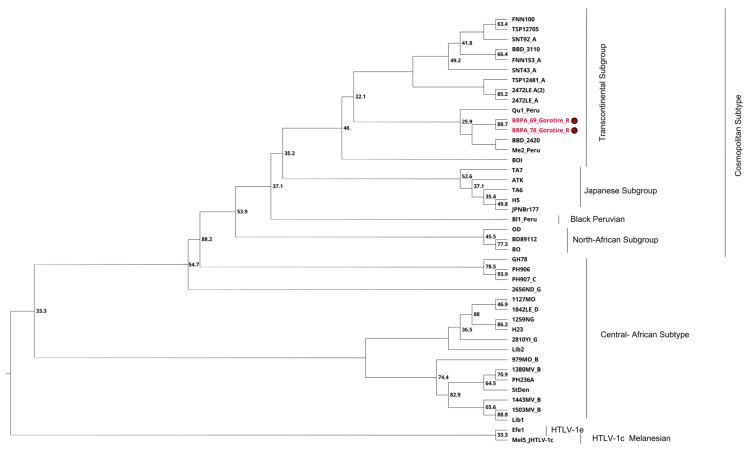
Rooted phylogenetic tree showing the relationships between the HTLV-1 samples available in GenBank and those described in the present study (BRPA_78_Gorotire_R; BRPA_69_Gorotire_R; highlighted in red). The tree was constructed using the maximum likelihood method after partial alignment of nucleotides in the 5‘LTR-1 region. The statistical sustainability test (bootstrap) was applied with 1000 replicates from the sequence bank.

**Figure 4:**
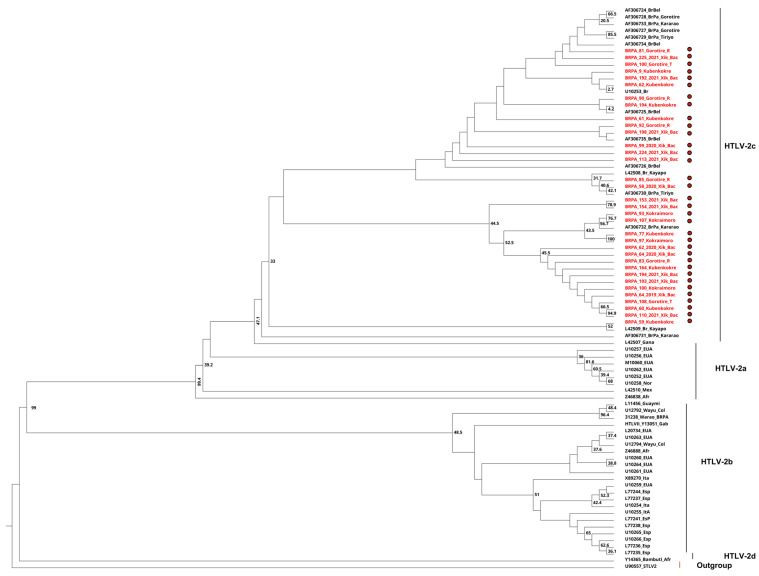
Rooted phylogenetic tree showing the relationships between the HTLV-2 samples available in GenBank and the 35 samples (highlighted in red) described in the present study. The tree was constructed using the maximum likelihood method after partial alignment of nucleotides in the 5‘LTR-2 region. The statistical sustainability test (bootstrap) was applied with 1000 replicates from the sequence bank.

In the 38 families investigated, due to funding limitations and the quality of the sequences obtained, it was possible to sequence samples from 13 family groups and three individuals without intrafamily transmission. Of the samples positive for HTLV-2, 16.3% (45/269 samples) were subjected to amplification of the 5′LTR region; however, amplification was not successful in 10 of the 45 samples. On the other hand, 100% of the samples confirmed to harbor HTLV-1 (*n* = 2) were successfully sequenced. Thus, 35 samples positive for the 5′LTR region of HTLV-2 and two positive for HTLV-1 were sequenced. The phylogenetic analysis of the two samples confirmed to be positive for HTLV-1 showed grouping to the cosmopolitan subtype (HTLV-1a) and transcontinental subgroup (A), supported by low bootstrap values of 53.9% and 46%, respectively (Figure 3), while all 35 samples positive for HTLV-2 were grouped as subtype 2c, with a low bootstrap value of 33% (Figure 4).

Among the 13 families whose samples were sequenced, similarity analysis of the nucleotide sequences of the 5′LTR region was performed for individuals from seven family groups, each with two or three sequenced samples, as shown in Table 5. Among these, the lowest degree of similarity was 60%, which was found in families 18 and 35, as viral sequences were compared in the grandfather vs. granddaughter and the father vs. daughter, respectively. Particularly for these two families, a probable vertical transmission was suggested, considering the seropositivity of the mothers for HTLV-2, although it was not possible to obtain the sequences of their infecting viruses. Analysis of the HTLV-1 sequences revealed that the sequences of BRPA_78_Gorotire_R and BRPA_69_Gorotire_R had 99% similarity, strongly suggesting vertical transmission in family 38. In family 20, samples from a couple showed 99% nucleotide similarity, suggesting sexual transmission. In family 24, samples from a woman and her two children showed a similarity of 87%; however, among the sequences obtained from the brothers, the identity was 99%, which may suggest vertical mother–child transmission or cross-breastfeeding by another positive woman. The results for family 11 revealed vertical transmission between the mother and daughter; however, there is also a possibility of sexual transmission, as the 25-year-old daughter reported not using a condom with her monogamous partner. Finally, in family 29, given that the similarity between the samples from a couple was 74%, it is possible that both had already been infected before becoming involved. For the remaining samples, in two family groups, only one sample from each family was sequenced (families 22 and 27).

**Figure 5:**
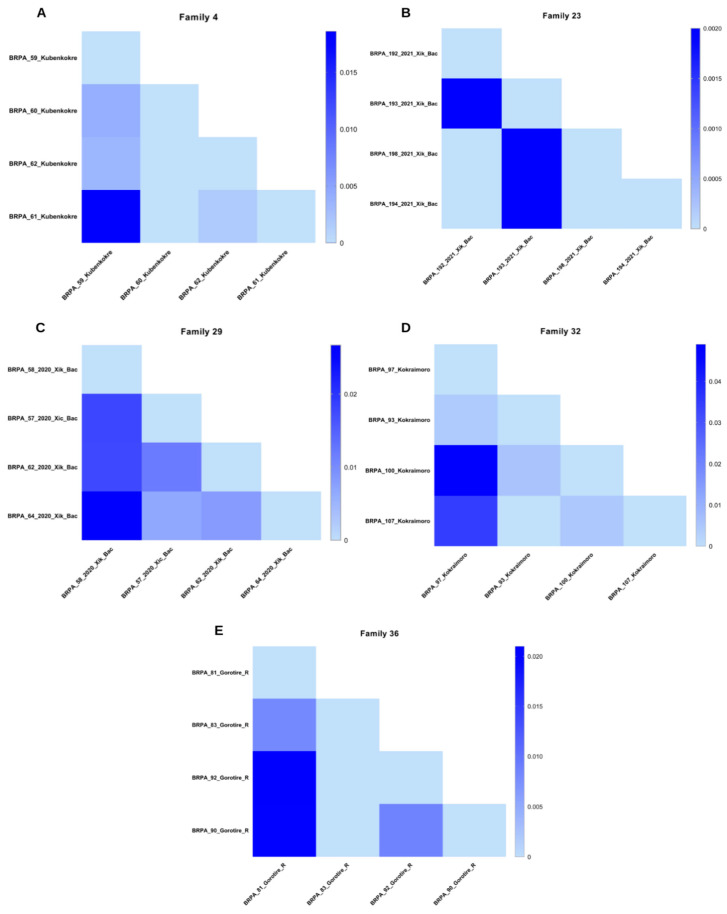
Heatmap showing the distance matrix between the nucleotide sequences of the 5‘LTR region of HTLV-2 in the five family groups. (**A**) family 4; (**B**) family 23; (**C**) 29; (**D**) family 32 and (**E**) family 36.

For the remaining five families, the genetic distance matrix was calculated by comparing the sequenced samples from families 4, 23, 29, 32, and 36 (Figure 5). Among the five families analyzed, we observed that in family four (Figure 5A), the largest distance of 0.0186 was identified between the samples of a positive grandmother (BRPA_59_Kubenkokre) and her grandson (BRPA_61_Kubenkokre). In family 23 (Figure 5B), the distances were greater (0.0020) between the spouses’ samples (BRPA_192_2021_Xik_Bac x BRPA_193_2021_Xik_Bac), just as the father’s sample also presented the same distance value compared with the samples from the two positive daughters. In family 29 (Figure 5C), the grandfather (BRPA_58_2020_Kik_Bac) and granddaughter (BRPA_64_2020_Xik_Bac) samples were the most distant (0.0267) compared with the grandmother and granddaughter samples (0.0066). For family 32 (Figure 5D), the distance between the mother (BRPA_97_Kokraimoro) and son (BRPA_100_Kokraimoro) was the greatest (0.0490), unlike that (0.0061) between the son (BRPA_100_ Kokraimoro) and his wife (BRPA_93_Kokraimoro). Finally, in family 36 (Figure 5E), the greatest distances observed were between the samples of the father and his daughters (0.0210).

Among families 16, 18, 22, 26, 29, and 32, eight individuals were identified whose parents and also spouses were positive. In these cases, two transmission hypotheses (vertical and sexual) were suggested. Through genetic distance analyses, we suggest that in family 29, the 50-year-old woman whose parents were positive, as well as her spouse, was possibly infected sexually, as indicated by the highest genetic distance value (0.0117) in relation to his mother’s sample. In this same family, it was possible to observe the transmission of the virus to the third generation, probably through breastfeeding, accounting for the observed genetic distance (0.0015). Unfortunately, it was not possible to compare the sequences with those of the husband‘s sample, as the sample was not amplified by PCR, making sequencing impossible. In family 32, from the Kokraimoro group, the four sequenced samples showed the greatest distances compared with those of samples from other peoples. The 49-year-old man whose wife and mother were also positive showed greater genetic distance from his mother‘s sample (0.0490) than from his wife‘s sample (0.0061). The samples from his wife and daughter strongly suggested vertical transmission since the distance was 0.0000.

**Figure 6:**
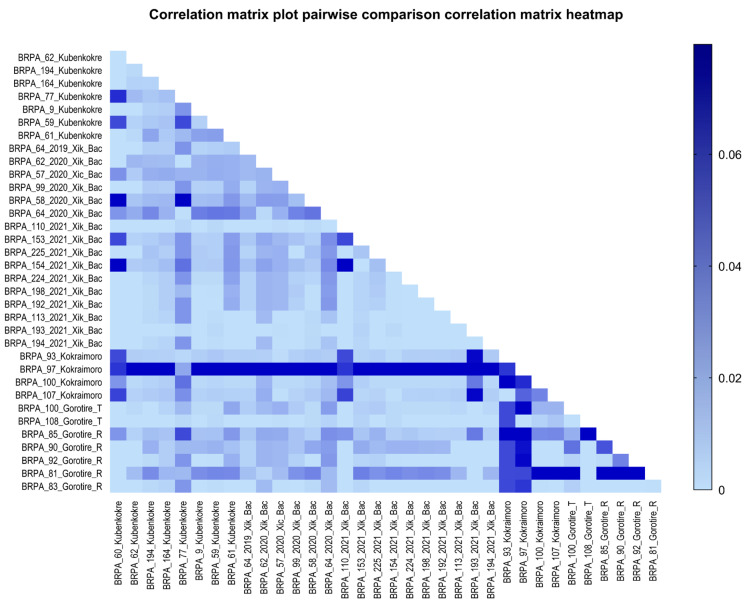
Heatmap showing the comparison between the samples sequenced for HTLV-2 in this study.

**Figure 7:**
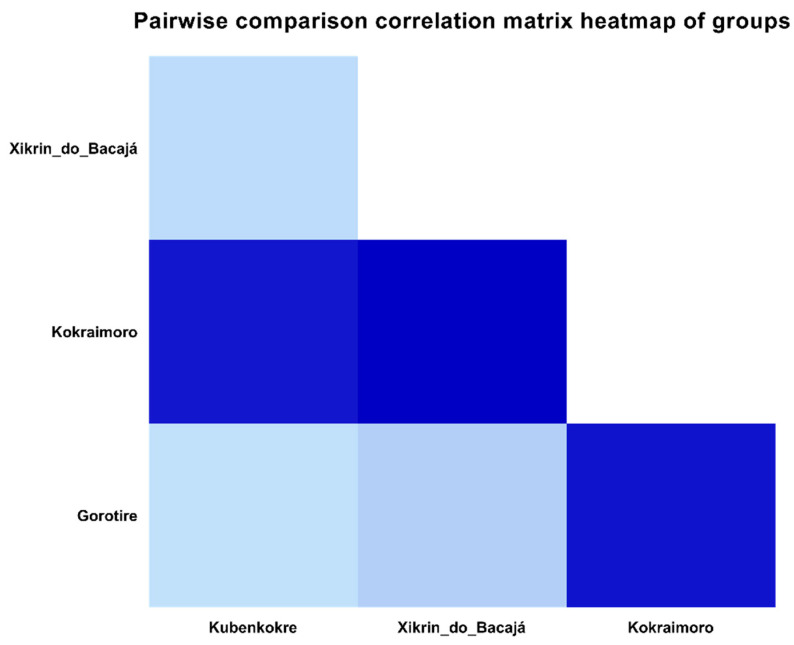
Heatmap showing the comparison between samples sequenced for HTLV-2 among four indigenous peoples (Gorotire, Kokraimoro, Kubenkokre, and Xikrin do Bacajá).

According to the analysis of the 35 sequenced samples, considering that all the HTLV-2 samples belonged to subtype 2c, most of the sequences presented a high degree of similarity, with an average distance of 0.0082, with 0.0796 being the greatest distance found (Figure 6). When comparing the sequences of four peoples (Gorotire, Kokraimoro, Kubenkokre, and Xikrin do Bacajá), despite the great similarity between the nucleotide sequences, we observed greater genetic distances between the samples from the Kokraimoro community and those from the other groups, i.e., the Xikrin people of Bacajá (0.0162), Gorotire (0.0149) and Kubenkokre (0.0148) (Figure 7).

## 4. Discussion

HTLV-2 infection is considered endemic among intravenous drug users [36,56,57] and among Indigenous peoples in the Americas, Europe, and Central Africa [49,50,58,59]. HTLV-1 infection, on the other hand, has a wider geographic distribution and is endemic in Africa, Asia, South America, the Caribbean, and the Australo-Melanesian region [45]. In Brazil, the prevalence varies by state, reaching rates above 1%, as observed in the population of Salvador (Bahia), which is the Brazilian state with the highest number of cases, with a prevalence of 1.7% [60]. In indigenous peoples in the state of Pará, especially in those belonging to the Kayapó ethnic group, the rates vary from 32.3% to 33% [48,49].

Although for accurate comparison of the prevalence of infection between studies conducted in different populations, it is important to analyze data by age group, sex, and sample size, our results reaffirm the high endemicity of HTLV-2 among indigenous peoples, especially among the Kayapó people (18.5%). However, the lower prevalence in this study can be explained by the larger sample size investigated (*n*= 1452) compared to previous studies, as well as the result of population fission events, which are very common among these people and can result in the dispersion of the infection at low frequencies among newly formed villages. For HTLV-1 infection, the 0.1% prevalence described herein is similar to that reported by Ishak et al. [49], who reported 0.48% infection among the Kayapó, as well as the results of Amianti et al. [61], who described a prevalence of 0.1% in indigenous people from a reserve in the state of Mato Grosso do Sul. The presence of this viral type in the Indigenous population is the result of contact between these people and neighboring nonindigenous populations, mainly through socioeconomic activities, mostly carried out by men from the village, as suggested by Abreu et al. [50] and Ishak et al. [59].

The main routes of transmission and spread of the virus among indigenous groups are sexual and vertical, especially through breastfeeding, as observed by Biglione et al. among Motaco indigenous people in Argentina [62]. Analysis of family groups revealed an intrafamily transmission rate of 42.7%, similar to that found (36.3%) by Silva et al. [63] in family groups of patients infected with HTLV-1 and that (43.5%) reported by Costa et al. [64] in family members of patients with HTLV-1/2 who were receiving outpatient care. Among family members of drug users, intrafamily transmission is even greater, reaching 61.4% [37].

In our study, among the suggested transmission routes, vertical transmission was the most frequent (47.4%). However, no significant difference was detected (*p* = 0.968) when comparing the efficiency of vertical transmission to sons and daughters, as also reported by Costa et al. [64]. Liu et al. [65] demonstrated that the vertical transmission route is more likely. However, the sexual transmission route was observed in 15.8% of the family groups we studied, which is lower than the prevalence described by Pereira et al. [66] (71.4%) and by Oliveira-Filho et al. [37] (76.9%). The differences between the transmission routes described in the literature and those observed in this study can be explained by the large number of children that Indigenous women have, as well as by the practice of cross-breastfeeding, which increases the transmission of the virus among children in the village.

Regarding the two unusual cases observed in family 13, although the mother was not tested, it is possible that the virus spreading occurred to the three positive children (aged 6, 10, and 13 years old) through direct vertical transmission by the mother or through cross-breastfeeding. In family 7, for the two positive daughters, aged 21 and 27 years old, whose father was positive and whose mother was negative, we suggest that the transmission occurred by cross-breastfeeding or sexual transmission.

In this study, we identified 25 children younger than 10 years who were infected. Considering that 98.3% of the positive women stated that they had breastfed their children for more than six months, we suggest that this transmission occurred through breastfeeding from mother to child or through cross-breastfeeding, corroborating previous results by Ishak et al. [32].

An increase in the prevalence of infection was observed with age in both sexes; however, women over 40 years of age were the most affected, corroborating other studies that described a higher prevalence of infection among women due to the greater transmission efficiency from men to women in sexual relationships [28,67].

Regarding blood transfusions reported among positive participants, only two (2/6) reported that they received a transfusion, one in 1963 and another in 1997, the first of which was before the implementation of screening for HTLV in blood banks in Brazil [42]. Because we did not observe intrafamilial transmission among these individuals, the infection may have occurred via blood transfusion, considering that the state of Pará has a high prevalence of the virus in blood donors [40,68].

Genetic similarity analyses strongly suggest the vertical transmission route in two cases: between a mother and her daughter and between a couple (with 99% similarity between the nucleotide sequences for the LTR region in both cases). This result can be explained by the lack of condom use in this population, which was reported by the vast majority of participants. A third case also stands out, involving a positive couple who may have acquired the infection through sexual transmission or previous mother-to-child infection, as low similarity was observed between the samples (74%).

Among the genetic distance analyses, we noticed that the greatest intrafamily distances occurred between the samples of parents and children. Significant distances also occurred between grandparents and grandchildren. We also noted that the lowest genetic distance values were between the samples of mothers and children, reinforcing the hypothesis of vertical transmission as the route of dissemination of HTLV-1 and 2.

Molecular characterization studies of HTLV-1 have classified it into seven viral subtypes according to the geographic region of origin, known as a, b, c, d, e, f, and g, with the cosmopolitan subtype (1a) being the most widely distributed in the world. This subtype is further classified into five subgroups, A, B, C, D, and E, the first of which (1aA) is called transcontinental [69,70,71]. However, HTLV-2 is classified into four viral subtypes, among which subtypes 2a and 2b are distributed throughout the Americas, subtype 2c is found exclusively in the Brazilian Amazon, and subtype 2d is restricted to Central Africa [49,72,73,74]. The samples analyzed in this study were grouped into the cosmopolitan 1a subtype and transcontinental subgroup (1aA), as were those in other studies carried out in the state of Pará [75,76]. The low prevalence found in this study (0.1%) corroborates the study by Ishak et al. [49] and suggests that the virus entry route may involve contact with a nonindigenous population, mainly miners who are present in the region close to the Gorotire people. For HTLV-2, phylogenetic analysis confirmed the presence of a molecular variant called subtype 2c, as previously described [49,72,73,74,77].

In conclusion, the present study confirmed intrafamilial transmission, especially vertical transmission, as one of the fundamental ways of the viral spread in Indigenous peoples, given that breastfeeding is one of the main ways of feeding children, which contributes significantly to the high endemicity of the virus. Sexual transmission was also suggested for some family groups. However, it is important to highlight the complexity of the process of the interrelationship between individuals within these communities; for many of them, endogamy and polygamy have been a cultural reality for generations, as well as the cross-breastfeeding, which can be confounding factors in defining the real route of intrafamilial transmission of HTLV in Indigenous peoples.

Furthermore, we confirmed the endemic nature of the HTLV-2c subtype among the indigenous peoples of the Brazilian Amazon, as well as the presence of the HTLV-1aA subtype, in accordance with research that previously described this virus as having a cosmopolitan character with wide distribution.

Notably, these results may be underestimated, as it is not possible to precisely state the number of families studied due to the difficulties in obtaining this information through demographic censuses in each indigenous territory. Health agencies are often unable to locate Indigenous people who frequently move to other villages, making it difficult to monitor information on each family.

In this context, it is crucial to implement new screening recommendations for HTLV-1 and HTLV-2 in pregnant women and newborns in Indigenous territories, and in cases of seropositivity, the suspension of breastfeeding must be carried out, thus interrupting one of the main routes of transmission of the virus HTLV-1/2.

## Figures and Tables

**Table 1:** Prevalence of HTLV-1 and HTLV-2 in five indigenous groups of the Kayapó people, Brazilian Amazon.

			qPCR				Western Blot					INNO-LIA					Total (%)				
People	N	EIA +	HTLV-1	HTLV-2	Neg	No	HTLV-1	HTLV-2	HTLV	Ind	Neg	HTLV-1	HTLV-2	HTLV	Ind	Neg	HTLV-1	HTLV-2	HTLV	Ind	Neg
Gorotire	474	108	2	99	6	1	0	6	1	-	-	-	-	-	-	-	2 (0.4)	105 (22.1)	1 (0.2)	-	-
Kararaô	39	0	-	-	39	-	-	1	-	3	11	-	-	-	-	24	-	1 (2.6)	-	3 (7.7)	35 (89.7)
Kokraimoro	140	18	-	14	3	1	-	2	1	1	-	-	-	-	-	-	-	16 (11.4)	1 (0.7)	1 (0.7)	-
Kubenkokre	398	93	-	85	6	2	-	7	-	1	-	-	-	-	-	-	-	92 (23.1)	-	1 (0.2)	-
Xikrin	401	61	-	45	15	2	-	3	2		-	-	7	1	3	1	-	55 (13.7)	3 (0.7)	3 (0.7)	1 (0.2)
Total	1.452	280	-				-	-	-	-	-	-	-	-	-	-	2 (0.1)	269 (18.5)	5 (0.3)	8 (0.5)	36 (2.5)

EIA: ELISA; Neg: negative; Ind: indeterminate; No: no material for DNA extraction.

**Table 2:** Prevalence of HTLV-1 and HTLV-2 infection by age and sex.

Age (Years)	Males		Females		Total Positives (%)
	Number	Positives	Number	Positives	
0–10	157	12	181	13	25/338 (7.4)
11–20	118	5	186	19	24/304 (7.9)
21–30	105	12	194	31	43/299 (14.4)
31–40	88	19	96	28	47/184 (25.5)
41–50	53	10	90	31	41/143 (28.7)
51–60	37	15	43	20	35/80 (31.2)
>61	49	23	55	38	61/104 (58.6)
Total	607	96	845	180	276/1452 (19.0)

**Table 3:** Risk factors associated with infection in four indigenous peoples, Kubenkokre, Kokraimoro, Gorotire, and Xikrin do Bacajá, among HTLV-1/2-positive and HTLV-2-negative individuals.

Risk Factors	Positive *n* = 192 (%)	Negative *n* = 569 (%)	*p* Values *
Piercing			0.6592
Yes	10 (5.2)	36 (6.3)	
No	179 (93.2)	515 (90.5)	
Unknown	3 (1.6)	18 (3.2)	
Blood transfusion			0.4493
Yes	6 (3.1)	25 (4.4)	
No	171 (89.1)	461 (81.0)	
Uninformed	15 (7.8)	83 (14.6)	
Breastfeeding as a child			0.2210
Yes	177 (92.2)	505 (88.8)	
No	4 (2.1)	24 (4.2)	
Uninformed	11 (5.7)	40 (7.0)	
First sexual intercourse			0.4991
≤17 years old	48 (25.0)	132 (23.2)	
≥18 years old	20 (10.4)	42 (7.4)	
Uninformed	124 (64.6)	390 (68.5)	
Did not start sex life	0	5 (0.9)	
Sexually active			1.0000
Yes	153 (79.7)	473 (83.1)	
No	32 (16.7)	77 (13.5)	
Uninformed	7 (3.6)	19 (3.4)	
Condom use	*n* = 153	*n* = 473	1.0000
Yes	3 (1.9)	17 (3.6)	
No	132 (86.3)	417 (88.2)	
Sometimes	7 (4.6)	26 (5.5)	
Uninformed	11 (7.2)	13 (2.7)	
Number of sexual partners	*n* = 153	*n* = 473	1.0000
1	140 (91.5)	436 (92.2)	
2	2 (1.3)	3 (0.6)	
3 or more	3 (2.0)	13 (2.8)	
Uninformed	8 (5.2)	21 (4.4)	

* Test G: Uninformed data were not considered for statistical analyses.

**Table 4:** Risk factors associated with infection in four indigenous peoples, Kubenkokre, Kokraimoro, Gorotire, and Xikrin do Bacajá, among HTLV-1/2-positive and HTLV-2-negative women.

Variables	% Total	% Positive
Gestational rate	85.9 (402/468)	87.1 (115/132)
Breastfeeding rate	97.0 (390/402)	98.3 (113/115)
Cross-breastfeeding rate	11.0 (43/390)	15.0 (17/113)
Number of pregnancies	*n* = 402	*n* = 115
1–3	51.0 (205)	35.6 (41)
4–6	31.1 (125)	42.6 (49)
7 or more	17.7 (71)	20.9 (24)
Uninformed	0.2 (1)	0.9 (1)
Breastfeeding time	*n* = 390	*n* =113
Less than 6 months	96.2 (375)	2.6 (3)
6 months or more	2.3 (9)	95.6 (108)
Uninformed	1.5 (6)	1.8 (2)

**Table 5:** Comparison of nucleotide analyses of the 5‘LTR region between HTLV-1- and HTLV-2-positive samples from seven indigenous families.

Families	Samples	% Similarity	Probable Routes of Transmission	Degree of Kinship
11	BRPA_194_Kubenkokre x BRPA_164_Kubenkokre	85%	Vertical	Mother-daughter
18	BRPA_77_Kubenkokre x BRPA_9_Kubenkokre	60%	Vertical *	Grandfather—granddaughter
20	BRPA_153_2021_Xik_Bac x BRPA_154_2021_Xik_Bac	99%	Sexual	Couple
24	BRPA_224_2021_Xik_Bac x BRPA_225_2021_Xik_Bac	99%	Vertical	Brethren
	BRPA_64_2019_Xik_Bac x BRPA_225_2021_Xik_Bac	87%	Vertical	Mother-daughter
	BRPA_64_2019_Xik_Bac x BRPA_224_2021_Xik_Bac	87%	Vertical	Mother-son
29	BRPA_58_2020_Xik_Bac x BRPA_57_2020_Xic_Bac	74%	Sexual or vertical	Couple
35	BRPA_100_Gorotire_T x BRPA_108_Gorotire_T	60%	Vertical *	Father-daughter
38	BRPA_69_Gorotire_R x BRPA_78_Gorotire_R	99%	Vertical	Mother-son

* Probable vertical transmission was suggested, considering the seropositivity of the mothers for HTLV-2.

## Data Availability

The data analyzed in this study are included in the paper.
